# Innate immune sensing of coronavirus and viral evasion strategies

**DOI:** 10.1038/s12276-021-00602-1

**Published:** 2021-05-06

**Authors:** Yusuke Kasuga, Baohui Zhu, Kyoung-Jin Jang, Ji-Seung Yoo

**Affiliations:** 1 Department of Immunology, Hokkaido University Graduate School of Medicine, Sapporo, 060-8638 Japan; 2 Department of Pathology, School of Medicine, Institute of Biomedical Science and Technology, Konkuk University, Chungju, 27478 Republic of Korea

**Keywords:** Infection, Pattern recognition receptors, Immune evasion

## Abstract

The innate immune system is the first line of the host defense program against pathogens and harmful substances. Antiviral innate immune responses can be triggered by multiple cellular receptors sensing viral components. The activated innate immune system produces interferons (IFNs) and cytokines that perform antiviral functions to eliminate invading viruses. Coronaviruses are single-stranded, positive-sense RNA viruses that have a broad range of animal hosts. Coronaviruses have evolved multiple means to evade host antiviral immune responses. Successful immune evasion by coronaviruses may enable the viruses to adapt to multiple species of host organisms. Coronavirus transmission from zoonotic hosts to humans has caused serious illnesses, such as severe acute respiratory syndrome (SARS), Middle East respiratory syndrome (MERS), and coronavirus disease-2019 (COVID-19), resulting in global health and economic crises. In this review, we summarize the current knowledge of the mechanisms underlying host sensing of and innate immune responses against coronavirus invasion, as well as host immune evasion strategies of coronaviruses.

## Coronaviruses: Improved understanding of immune system responses needed

Understanding how the innate immune system senses coronaviruses and how coronaviruses can escape detection could provide novel approaches to tackle infections. Coronaviruses, including SARS-CoV-2, constantly evolve to manipulate, obstruct and evade host immune responses. A team led by Ji-Seung Yoo, Hokkaido University, Sapporo, Japan, reviewed understanding of innate immune responses to coronaviruses and viral evasion strategies. Two major receptor families recognise RNA viruses upon infection, but how they respond to SARS-CoV-2 is unclear. One receptor, TLR7, plays a critical role in sensing coronavirus infections, and mutations in the *TLR7* gene are associated with severe illness and mortality in young Covid-19 patients. Activating host TLR pathways may prove a useful therapeutic approach. Further in-depth investigations are needed into specific coronavirus proteins and viral mechanisms that suppress host immunity.

## Introduction

Protecting the ‘self’ from the ‘non-self’ is essential for maintaining life. Therefore, distinguishing between self and non-self is critical. In living organisms, the invasion of harmful factors can be recognized and protected against by two systems: biological barriers, such as skin and mucous membranes, and host immunity. In some organisms, including humans, the latter can be further divided into innate and acquired immunity. Innate immunity, along with biological barriers, constitutes the first line of defense against pathogen infection. Activation of the innate immune system is initiated by the recognition of harmful factors, such as pathogen-associated molecular patterns (PAMPs) and damage-associated molecular patterns (DAMPs), through germline-encoded pattern recognition receptors (PRRs). Activated PRRs rapidly trigger multiple host defense programs to protect against harmful invaders, such as viruses. Therefore, the innate immune system is frequently targeted by pathogens for immune evasion. Dysregulation or imbalance of innate immune responses induced by pathogens often leads to the onset of various diseases.

Coronaviruses have evolved multiple viral strategies to evade host antiviral immunity. Although coronaviruses are recognized as highly virulent pathogens in veterinary medicine, they have been treated as mild infectious agents that cause a seasonal cold with mild respiratory illness in humans. However, some coronavirus strains of zoonotic origin, such as severe acute respiratory syndrome-associated coronavirus (SARS-CoV), Middle East respiratory syndrome coronavirus (MERS-CoV), and the recently emerged SARS-CoV-2, have caused repeated outbreaks, infecting humans and threatening human health since the first outbreak of SARS-CoV in 2003. These coronaviruses perturb the host defense program by impairing antiviral innate immune responses through multiple viral inhibitory mechanisms, resulting in successful viral transmission and adaptation to the human host.

Coronavirus disease-2019 (COVID-19) is a highly contagious serious respiratory disease that is caused by SARS-CoV-2 and was first identified in Wuhan, China, in December 2019. Since the outbreak was reported, SARS-CoV-2 has rapidly spread across the globe, and the World Health Organization declared a pandemic of SARS-CoV-2 on 11 March 2020^1^. The COVID-19 pandemic has drastically changed people’s lifestyles and placed great pressure on the current medical care system, with a tremendous social and economic burden. The existence of a myriad of coronaviruses in bats, including many SARS-related CoVs, and the sporadic crossing of coronaviruses over the species barrier to humans suggest that future occurrences of zoonotic transmission events may occur. Therefore, a solution for the control of the COVID-19 pandemic, as well as potential similar pandemics in the future, is urgently needed, especially for the prevention of viral spread and the treatment of virus-induced diseases. Understanding the underlying mechanisms of coronavirus-host interactions is critical for developing effective vaccines and therapeutics.

### Coronavirus

Coronaviruses belong to the *Orthocoronavirinae* subfamily of the *Coronaviridae* family. Viruses in the *Orthocoronavirinae* subfamily can be further classified into four genera: *Alphacoronavirus*, *Betacoronavirus*, *Gammacoronavirus*, and *Deltacoronavirus*^2^. Among these viruses, seven strains of *alpha*- and *betacoronaviruses* are known to cause human illness. HCoV-229E, HCoV-OC43, HCoV-NL63, and HCoV-HKU1 cause mild upper respiratory symptoms that are typically recognized as the seasonal common cold^2^. In contrast, the other three strains, SARS-CoV, MERS-CoV, and SARS-CoV-2, which are transmitted through zoonotic transmission, can lead to severe respiratory symptoms with unique pathogenesis, including severe lymphopenia and extensive pneumonia caused by aberrant host antiviral responses^3^.

Coronaviruses are enveloped positive-sense RNA viruses that possess the largest viral RNA genome, ~27–32 kb in size. Upon coronavirus infection, the virus life cycle initiates with the specific binding of virus spike (S) protein to the target cell surface receptors, causing viral fusion into the target cell^4^. During the intracellular viral life cycle, the genomic RNA of coronavirus is uncoated from the nucleocapsid (N) protein, leading to the translation of two open reading frames (ORFs), ORF1a and ORF1b. The translated ORFs produce two large polyproteins, pp1a and pp1ab, which are further cleaved by viral proteases encoded by the Nsp3 (papain-like protease) and Nsp5 (3C-like protease) genes to generate functional nonstructural proteins (Nsp1-Nsp16)^5^. The viral replication and transcription complex (RTC) formed by Nsp2-Nsp16 further promotes viral genomic RNA replication and subgenomic mRNA transcription. Among these proteins, Nsp3, Nsp4, and Nsp6 are involved in double-membrane vesicle (DMV) formation, together with two other viral replication organelles, namely, convoluted membranes (CMs) and small open double-membrane spherules (DMSs), providing a protective microenvironment for the replication of viral genomic RNA and transcription of subgenomic mRNAs^6^. Nsp7 and Nsp8 are two cofactors of RNA-dependent RNA polymerase (RdRp) that reside in Nsp12^7^, and together with RNA-modifying enzymes residing in Nsp13-Nsp16, they promote viral RNA synthesis and modification^8^. Notably, a 3′–5′ exonuclease residing in Nsp14 has an RNA proofreading function during RNA synthesis^9^. The RNA capping machinery during the viral life cycle is formed by Nsp10 (cofactor), Nsp13 (RNA 5′ triphosphatase activity), Nsp14 (N7-methyltransferase activity), and Nsp16 (2′-O-methyltransferase activity), while the mechanism remains to be elucidated^10–12^. Moreover, additional accessory proteins, including ORF3, 6, 7, 8, 9, and 10, with various host-modulatory functions are also necessary for completion of the viral life cycle^13^. The exact number, location, and size of accessory proteins vary in different coronaviruses, and their specific functions remain to be further elucidated. Structural proteins such as S, envelope (E), membrane (M), and N, translated from subgenomic mRNAs, translocate from the endoplasmic reticulum (ER) to the ER-to-Golgi intermediate compartment (ERGIC) and assemble with newly generated genomic RNA encapsidated by the N protein. Mature virions are released by the host exocytosis pathway to target the next host cell^14^.

### Host innate immune sensors

Immediate cellular responses to pathogen invasion are crucial for maintaining cell homeostasis and survival for all living organisms. Host responses are triggered by germline-encoded cellular receptors, known as ‘pattern recognition receptors’ (PRRs), that recognize specific patterns of ‘non-self’ and ‘danger’ molecules, termed ‘pathogen-associated molecular patterns’ (PAMPs) and ‘danger-associated molecular patterns’ (DAMPs). In mammals, activation of PRRs by PAMPs or DAMPs triggers innate immune responses and produces multiple IFNs and proinflammatory cytokines. In recent decades, various PRRs, such as Toll-like receptors (TLRs), nucleotide-binding oligomerization domain (NOD)-like receptors (NLRs), C-type lectin receptors (CLRs), AIM2-like receptors (ALRs), cyclic GMP-AMP synthase (cGAS), and retinoic acid-inducible gene I (RIG-I)-like receptors (RLRs), have been discovered^15–19^. Among these receptors, TLRs and RLRs are two major receptors responsible for sensing RNA virus infection and triggering antiviral IFN programs.

**Fig. 1:**
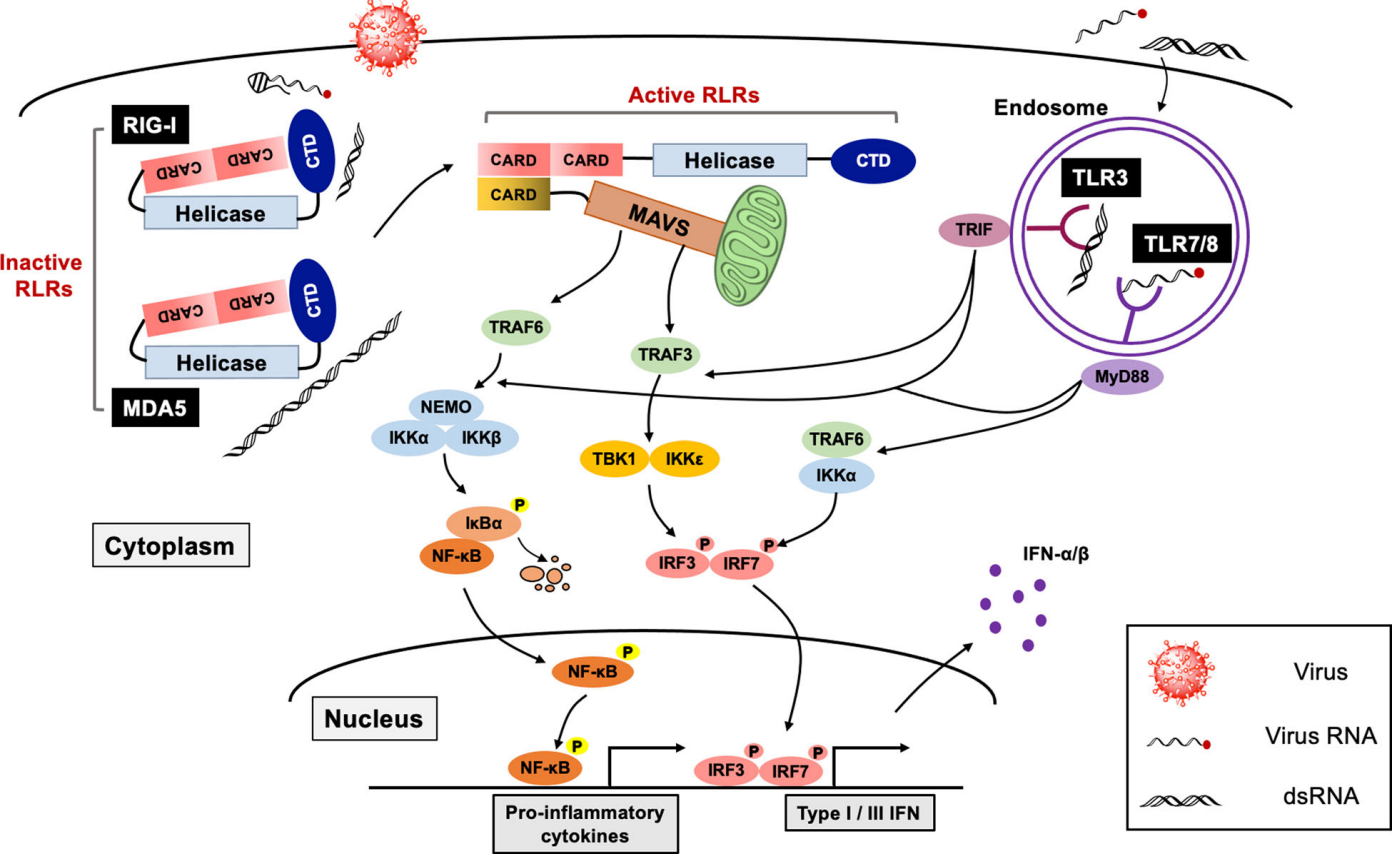
Sensing of RNA virus invasion by RLRs and TLRs. Invasion by RNA viruses is sensed by cytosolic and endosomal RNA sensors, RLRs and TLRs. Both RLRs and TLRs can detect viral RNA species such as viral genomic RNA and dsRNA produced during viral replication. While RIG-I and MDA5 are responsible for sensing cytoplasmic viral RNAs such as 5′-ppp RNA with a secondary structure (RIG-I) or dsRNA (short dsRNA; RIG-I and long dsRNA; MDA5), TLRs can detect endosomal ssRNA (TLR7/8) or dsRNA (TLR3). Activated RLRs undergo a conformational change that allows RLRs to expose CARDs and trigger the IFN signaling pathway through the CARD-CARD interaction between RLRs and MAVS. On the other hand, activated TLR3 and TLR7/8 initiate the antiviral IFN program by recruiting the signal adapter molecules TRIF and MyD88, respectively. Then, subsequent activation of the shared downstream kinases and transcription factors elicits the production of IFNs and proinflammatory cytokines.

Toll was first identified as an antifungal gene responsible for the Drosophila immune system, and later studies further elucidated the fundamental role of TLRs in innate immune sensing in mammals^20^. To date, multiple TLRs have been discovered, and their functions have been clarified. For example, 10 TLR members were identified (TLR1-TLR10) in humans, and 12 mouse TLRs (TLR1-TLR9 and TLR11-TLR13) were discovered, and their functions were examined^15,21^. Each TLR can recognize common or distinct PAMPs that are typically derived from components of microbes such as nucleic acids, lipoproteins, and lipids. Among the TLRs, TLR 3, 7, and 8 are responsible for recognizing RNA viruses entering through endocytosis by sensing single- or double-stranded RNA (ssRNA: TLR7/TLR8; dsRNA: TLR3) in endosomal compartments (Fig. 1).

Unlike TLRs, RLRs are essential cytoplasmic viral sensors that recognize intracellular non-self RNAs possessing distinct patterns of secondary structures or biochemical modifications^18,22^. RLRs are Asp-Glu-Ala-Asp (DEAD) box containing RNA helicases composed of three members, RIG-I, melanoma differentiation-associated gene 5 (MDA5), and laboratory of genetics and physiology 2 (LGP2). Structurally, all RLRs possess an RNA helicase domain with a C-terminal domain (CTD) that is responsible for RNA binding. While RIG-I and MDA5 have tandem caspase activation and recruitment domains (CARDs) for downstream signal transduction, LGP2 lacks a CARD domain, thereby functioning as a modulator of RIG-I and MDA5^23^. RIG-I and MDA5 can recognize non-self RNAs through common and distinct mechanisms. dsRNA is a classical non-self RNA that is not produced in uninfected cells due to a lack of RNA-dependent RNA polymerase in mammalian cells. Both RIG-I and MDA5 can be activated by an artificial dsRNA, polyinosinic:polycytidylic (poly I:C) acid. Intriguingly, activation of RIG-I and MDA5 by dsRNA is differentially regulated in a dsRNA length-dependent manner^24^. In addition to the double-stranded structure, recent studies have identified several biochemical properties of RLR-activating RNA species, such as (1) 5′-triphosphate with secondary structured RNAs^25^, (2) 5′-diphosphate uncapped RNAs^26^, and (3) RNAs with an unmethylated 5′-end nucleotide at the 2′-O position^27,28^, which are typically generated during replication of RNA viruses, including coronaviruses (Fig. 1).

Upon RNA ligand binding, RLRs and TLRs immediately initiate antiviral defense programs. TLRs initiate downstream signal cascades by recruiting adapter proteins, such as myeloid differentiation primary response 88 (MyD88) (for TLR7 and TLR8) and TIR-domain-containing adapter-inducing IFN-β (TRIF) (for TLR3). On the other hand, RLRs undergo conformational changes from inactive ‘closed’ to active ‘open’ structures mediated by ATPase/helicase activity. Activated RLRs liberate CARDs to bind to the signaling adapter molecule mitochondria antiviral signaling protein (MAVS) via the CARD-CARD interaction. The signaling adapters MyD88, TRIF, and MAVS then coordinate downstream signaling pathways by recruiting several ubiquitin ligases, such as TNF receptor-associated factor (TRAF) 3 and TRAF6, that associate with antiviral kinases, such as TANK-binding kinase 1 (TBK1), I-kappa-B kinase ε (IKKε), and the IKKα/β/γ complex. Consequently, activation of the transcription factors IRF3 and IRF7 and NF-κB leads to the production of type I IFN and proinflammatory cytokines to operate the host antiviral IFN programs^29^ (Fig. 1).

### Antiviral responses by IFN-inducible proteins

Viral recognition by the host innate immune system rapidly initiates the production of IFNs, triggering the expression of hundreds of ISGs to facilitate further antiviral responses. Subsequently, the produced IFNs and cytokines coordinate timely and balanced early immune responses that further provoke host antiviral defense programs by recruiting multiple types of immune cells to viral infection sites^30^. Binding of type I and type III IFNs to their cognate receptors, IFNAR1/IFNAR2 and IFNLR1/IL-10R2, initiates downstream antiviral signaling cascades by activating the associated tyrosine kinases JAK1 and TYK2^31^. Subsequent activation of the transcription factors signal transducer and activator of transcription (STAT) 1 and STAT2 by the kinases induces the formation of the IFN-stimulated gene factor 3 (ISGF3) complex (IRF9/p-STAT1/p-STAT2), which then acts as a transcription factor to drive the expression of interferon-stimulated genes (ISGs). Proteins encoded by ISGs play a crucial role in antiviral responses by targeting steps in the viral life cycle. For example, interferon-inducible transmembrane (IFITM) proteins have been shown to inhibit viral entry by blocking viral envelope fusion with cellular membranes^32^ or suppressing the intracellular trafficking of incoming viral particles^33,34^. The IFN-inducible IFI16 protein, an innate immune sensor of intracellular DNA, can downregulate viral mRNA synthesis^35^. Protein kinase dsRNA-activated (PKR), a cytoplasmic double-stranded RNA sensor, plays a role in the antiviral response by inhibiting protein translation and inducing cell apoptosis^36^. Human IFIT family proteins have also been reported to inhibit viral protein production by binding to subunits of the eukaryotic initiation factor 3 translation complex by recognizing RNAs lacking 2′-O methylation^37^. Cellular enzymes, such as oligoadenylate synthetases (OAS) and ribonuclease RNase L, cooperatively play antiviral roles. Upon dsRNA recognition, OAS proteins catalyze the formation of 2′–5′ oligoadenylates (2–5 A), which subsequently activates RNase L, leading to degradation of all RNAs in the cell^38^. In the late stage of the viral life cycle, tetherin was shown to play a role in inhibiting viral release by hijacking budding virions on the cell surface^39^. Although the functions of many other ISGs have not been fully characterized, the timely expression and function of IFN-induced proteins are essential for protection against viral invasion.

## Sensing of coronavirus infection by host innate immune sensors

Host innate immune sensors can detect RNA virus infection by sensing ‘incoming’ viral genomes or ‘replication intermediate’ RNAs. During coronavirus replication, viral RdRp generates cytoplasmic PAMP RNAs. However, due to the viral immune evasion achieved by targeting host sensing pathways, type I and III IFN production is often abrogated by coronavirus infection. Nevertheless, there is evidence that multiple innate immune receptors are responsible for sensing coronavirus invasion (Table 1).

**Table 1:** Sensing of coronaviruses by the host antiviral sensors.

Sensor	Type of coronavirus	Refs.
RIG-I	MHV	^40^
	MERS	^43^
MDA5	MHV	^27,40–42^
	HCoV-229E,	^27^
	MERS	^43^
TLR2	SARS-CoV-1	^54^
TLR3	SARS-CoV-2	^47^
	SARS-CoV-1	^48^
TLR4	SARS-CoV-1	^48^
	MHV	^49^
TLR7	MHV	^50^
	SARS-CoV-1	^50^
	MERS	^51^
	SARS-CoV-2	^52^
OAS/RNase L	MHV	^58^
PKR	MHV	^59^
	MERS	^60^
PACT	MERS	^61^
IFIT	MHV	^27^
	SARS-CoV-1	^62^

### Recognition of coronavirus by RLRs

Induction of type I IFN by mouse hepatitis virus (MHV), a murine coronavirus, is regulated by both RIG-I and MDA5 in brain cells^40^. In addition, MDA5 plays a pivotal role in controlling the pathogenesis of MHV^41^. HCoV-229E and MHV can evade MDA5 sensing through viral 2-O-methyltransferase, which sequesters viral RNAs^27^. Moreover, a recent study elucidated that MHV Nsp15, a viral endonuclease, removes a polyuridine-rich region in the negative-stranded viral RNA (PUN RNA) that would otherwise trigger an MDA5-mediated antiviral response^42^. Therefore, these studies indicate that MDA5 may be a predominant cytoplasmic sensor of coronavirus RNAs. Although RLRs seem to be dispensable for IFN production against MERS-CoV, both RIG-I and MDA5 contribute to proinflammatory responses in MERS-CoV-infected macrophages^43^.

It is still unclear whether RLRs are responsible for sensing SARS-CoV-2 infection. Recent studies have shown that although SARS-CoV-2 infection abolishes the type I and III IFN signaling pathways, proinflammatory responses are still robustly induced during SARS-CoV-2 infection^44,45^. Given that coronaviruses replicate in the cytoplasm and generate cytoplasmic PAMP RNAs such as replicate intermediate dsRNAs^46^, it is reasonable to assume that RLRs may contribute to the recognition of SARS-CoV-2 infection and the induction of proinflammatory responses. However, it needs to be elucidated whether SARS-CoV-2 indeed produces cytoplasmic PAMP RNAs during replication and, if so, which cytoplasmic RNA sensor(s) are responsible for recognizing PAMP RNAs generated by SARS-CoV-2.

### Recognition of coronaviruses by TLRs

In addition to RLRs, several TLRs play essential roles in coronavirus-induced innate immune responses. A recent study showed that inborn errors of TLR3- and IRF7-mediated type I IFN immunity were associated with disease severity and mortality in COVID-19 patients^47^. Moreover, mice lacking TRIF and translocating chain-associated membrane protein (TRAM), downstream adapter molecules of TLR3 and TLR4, showed more severe viral pathogenesis accompanied by increased mortality induced by SARS-CoV infection^48^. Another study also showed that TLR4-deficient mice were more susceptible to MHV1 infection than wild-type mice^49^, suggesting essential roles of TLR3 and TLR4 in antiviral responses against coronavirus infection.

Studies have also shown that TLR7 plays a critical role in the sensing of coronavirus infection in certain types of immune cells. In conventional dendritic cells (cDCs) and plasmacytoid dendritic cells (pDCs), the production of type I IFN and proinflammatory cytokines upon MHV and SARS-CoV infection is TLR7 dependent^50^. Furthermore, a subsequent study showed that type I and type III induction by MERS-CoV are regulated by TLR7 in pDCs^51^. Most importantly, a recent clinical study discovered that several natural mutations in the *TLR7* gene that cause ‘loss-of-function’ are associated with the severity and mortality of young COVID-19 patients, demonstrating that TLR7 sensing of SARS-CoV-2 is critical for the control of COVID-19 pathogenesis^52^. These results suggest that activation of TLR sensing pathways could be a potential therapeutic approach for COVID-19.

Interestingly, several coronavirus proteins appear to induce host innate immune responses via TLR-derived signaling pathways. It has been shown that the spike protein of SARS-CoV could induce IL-8 production by activating the mitogen-activated protein kinase (MAPK)-AP1 axis signaling pathway in lung epithelial and fibroblast cell lines^53^. Another group also showed that the SARS-CoV spike protein can be recognized by TLR2 and triggers proinflammatory responses in PBMCs^54^. Furthermore, it was also reported that the SARS-CoV membrane (M) protein could induce type-I IFN production through an unknown cytosolic sensing system that may be involved in a noncanonical TLR signaling pathway^55^. Although the exact mechanisms underlying these regulatory effects are still unclear, these reports suggest that coronavirus proteins can serve as potential agonists of TLRs.

### Recognition of coronaviruses by other host factors

Other host factors are also involved in facilitating the sensing of coronavirus invasion. PKR, RNase L, and OAS1 are representative IFN-inducible antiviral proteins that recognize cytosolic dsRNA^56,57^. Recent studies have shown that dsRNA produced by mutant MHV lacking endoribonuclease activates host antiviral responses through PKR and OAS1-RNase L^58,59^. Moreover, it was shown that MERS-CoV evades the PKR-mediated antiviral stress response by sequestering viral RNA by p4a^60^. PACT, a protein activator of PKR, was also shown to be involved in sensing MERS-CoV infection^61^. Thus, these reports suggest that cytosolic antiviral proteins contribute to coronavirus sensing.

Members of the IFN-induced protein with tetratricopeptide repeat (IFIT) family are well-studied antiviral proteins that restrict viral replication. IFITs play critical roles in antiviral responses upon coronavirus infection. Züst et al.^27^ showed that IFIT1 could suppress MHV replication by sensing viral RNAs lacking 2′-O methylation. Another group also showed that the depletion of IFIT1 and IFIT2 led to a significant increase in SARS-CoV replication^62^, providing evidence that IFIT family members participate in host innate sensing of coronavirus invasion.

## Innate immune evasion by coronaviruses

**Fig. 2:**
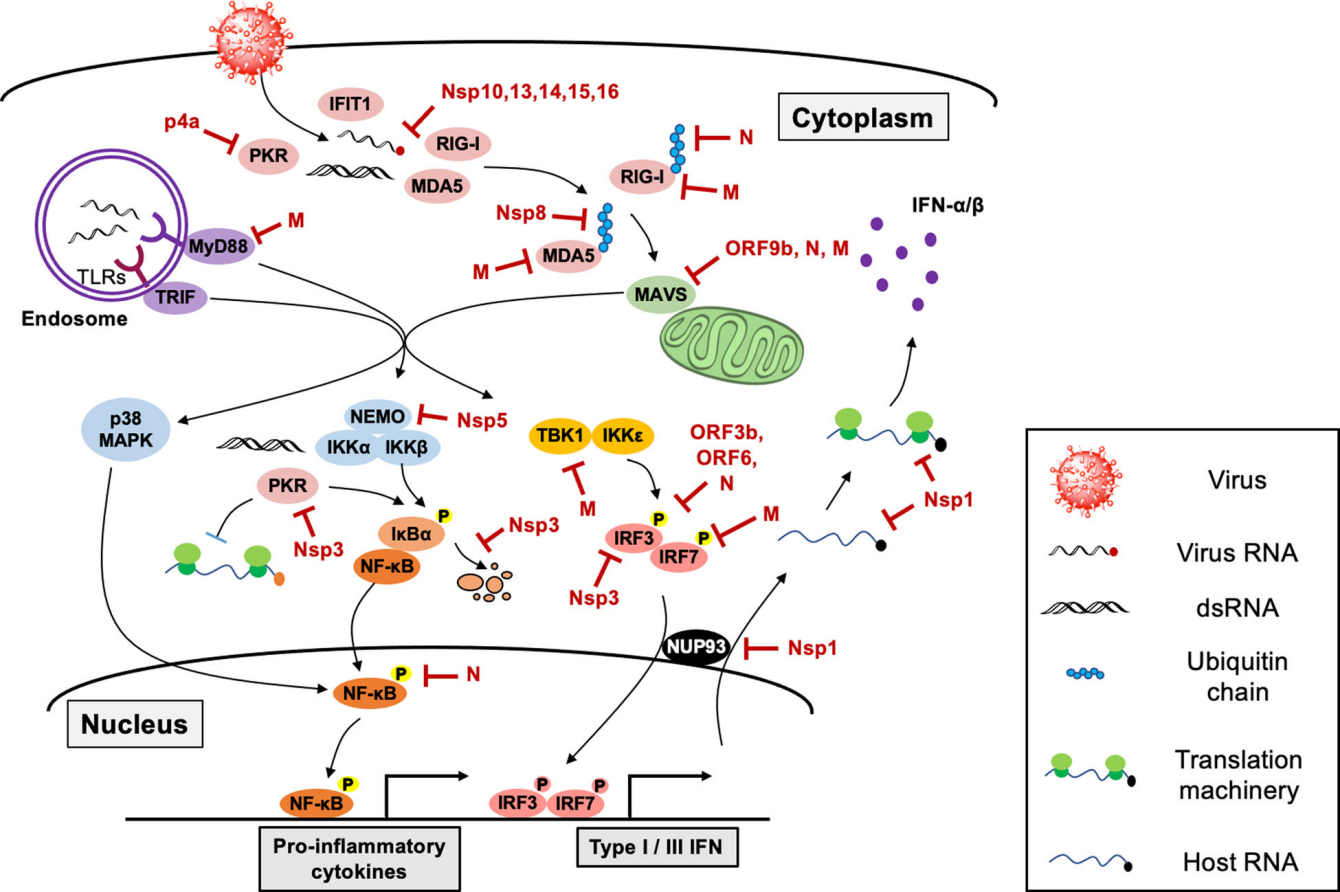
The host innate immune sensing pathway targeted by coronavirus. The multiple host factors in the antiviral signaling cascade are targeted by coronavirus proteins. Innate antiviral sensors can recognize coronavirus invasion by sensing cytosolic or endosomal viral RNA. As discussed, activation of virus sensors triggers an antiviral signaling cascade to elicit the production of type I or type III IFN as well as proinflammatory cytokines. On the other hand, coronaviruses have evolved multiple strategies to avoid host recognition by impeding the function of antiviral proteins using various viral proteins.

Although the host innate immune system possesses elaborate antiviral defense programs via unique and overlapping viral sensing mechanisms, viruses continuously develop new strategies to evade host antiviral defense programs. Most viruses utilize their proteins to antagonize the host innate immune system by either targeting viral sensors or blocking downstream antiviral signaling molecules. Likewise, coronaviruses have multiple strategies to hamper host innate immune responses using various viral proteins (Fig. 2 and Table 2). While coronaviruses that induce mild symptoms, such as HCoV-229E, provoke robust type I IFN production^63^, highly pathogenic coronaviruses that cause critical illness, such as SARS-CoV, MERS-CoV, and SARS-CoV-2^64^, have been shown to impede host antiviral programs. Thus, efficient immune evasion is closely associated with virulence and pathogenicity. A thorough understanding of the immune antagonizing mechanism used by these viruses is essential to design and develop effective antiviral therapeutics.

**Table 2:** Immune evasion strategies by coronavirus proteins.

Coronavirus protein	Immune evasion strategy	Refs.
Nsp1	Cleaves host mRNA, Inhibits protein translation, Suppresses function of STAT1 and c-jun	^65–71^
Nsp3	Inhibits type I IFN production, Suppresses ubiquitination and ISGylation, DMV formation	^72–77^
Nsp4	Sequesters viral RNA via DMV formation	^78^
Nsp5	Processes antiviral proteins	^79–81^
Nsp6	Sequesters viral RNA via DMV formation	^78^
Nsp8	Inhibits MDA5 activation	^82,83^
Nsp13	Remove 5′ ppp of viral RNA	^10,84,85^
Nsp14	RNA cap modification	^12,86^
Nsp15	Remove PUN RNA of viral RNA	^42^
Nsp16	RNA cap modification	^11,87^
ORF3a	Antagonizes IFN signaling, promotes apoptosis and inflammasome	^88–91^
ORF3b	Antagonizes IFN signaling	^92,93^
p4a (orf4a)	Antagonizes IFN signaling, antagonizes PKR function	^60^
ORF6	Inhibits nuclear transportation of antiviral proteins	^70,71,94–96^
ORF7a	Inhibits host protein translation, activate proinflammatory pathways	^97–100^
ORF9b	Processes antiviral proteins, antagonizes IFN signaling pathway	^101,102^
N	Suppresses RNA sensing pathways, inhibits function of STAT1/2	^103**–**105^
M	Inhibits TRAF3-TANK-TBK1/IKKε complex formation Inhibits viral sensing function of RIG-I and MDA5	^106**–**108^

### Nsp1

Nsp1 of coronaviruses has been reported to impede the host innate immune system by targeting multiple biological pathways. Nsp1 can inhibit protein translation by either blocking the assembly of the translation machinery or inducing the cleavage of 5′-capped host mRNAs. SARS-CoV Nsp1 blocks the translation of cap-dependent and internal ribosome entry site (IRES)-driven mRNA by binding to ribosomal subunits^65^. Similarly, another study also demonstrated that SARS-CoV-2 Nsp1 inhibits the translation of host antiviral proteins by targeting the 40S ribosomal subunit^66^.

Other studies have also shown that Nsp1 suppresses protein translation by inducing 5′-capped host mRNA cleavage. Intriguingly, SARS-CoV viral mRNAs, which contain an intact cap structure and poly-A tail, were not susceptible to Nsp1-mediated RNA cleavage^67^. Further analysis revealed that the presence of the 5′-end leader sequence in the viral mRNA could prevent Nsp1-mediated endonucleolytic RNA cleavage, indicating a unique immune evasion strategy by Nsp1^65,67^. Another novel inhibitory mechanism suggested is that SARS-CoV Nsp1 blocks mRNA nuclear export by directly inhibiting nuclear pore complex protein 93 (Nup93), thereby subsequently suppressing protein synthesis^68^.

**Fig. 3:**
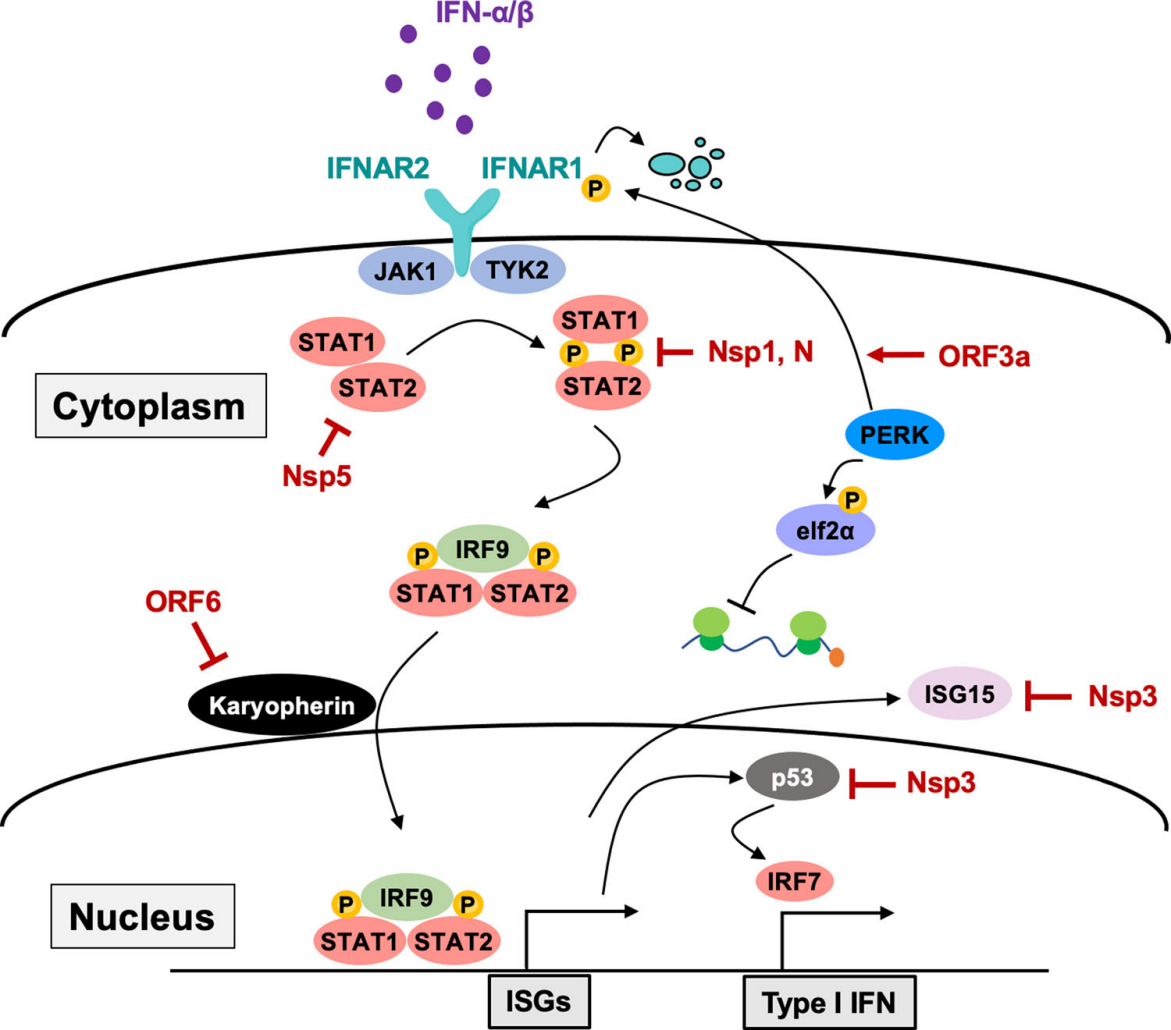
Antagonizing IFN-mediated immune responses by coronavirus. Secreted type I IFNs, IFN-α, and IFN-β can activate IFNAR1/2 in bystander cells to provoke the host antiviral program. On the other hand, coronavirus suppresses host antiviral IFN responses by targeting multiple components of the IFN signaling pathway through the inhibitory functions of viral proteins.

In addition to its inhibitory roles in protein synthesis, Nsp1 can also block the IFN signaling pathway (Fig. 3). Both virus- and IFN-induced antiviral responses were hampered by SARS-CoV Nsp1^69^. Moreover, recent studies have also shown that SARS-CoV-2 Nsp1 can strongly suppress the promoter activities of IFN-stimulated response elements (ISREs)^70,71^. Therefore, these reports provide evidence that Nsp1 has multiple antagonizing functions targeting host antiviral programs.

### Nsp3

Nsp3 is the largest protein among the genes encoded by the coronavirus genome and has multiple functions. Nsp3 plays a critical role in virus replication by processing the ORF1ab polyprotein via its ‘papain-like protease’ activity. Additionally, protease enzymatic activity appears to be involved in antagonizing the host innate immune response. SARS-CoV Nsp3 can bind to IRF3 and prevent the phosphorylation, dimerization, and nuclear translocation of IRF3, resulting in the suppression of the IFN signaling pathway^72^. Moreover, Nsp3 can inhibit the NF-κB signaling pathway by stabilizing IκBα, an NF-κB inhibitor^73^, suggesting that Nsp3 can inhibit host antiviral responses in a protease activity-independent manner.

Recently, accumulating evidence has suggested that the deubiquitinating (DUB) activity of Nsp3 is critical for inhibiting host IFN signaling pathways. Several studies have shown that Nsp3 antagonizes ubiquitination and ISGylation of host antiviral proteins, such as IRF3 and p53, resulting in suppression of antiviral responses^74–76^. Interestingly, the regulatory mechanism of the Nsp3 deubiquitinating function seems to be distinct between SARS-CoV and SARS-CoV-2. While SARS-CoV Nsp3 preferentially cleaves the ubiquitin chain of target proteins, ISGylated proteins are predominantly processed by SARS-CoV-2 Nsp3^75,77^, suggesting shared but distinct inhibitory strategies via Nsp3 of different coronaviruses. Thus, targeting Nsp3 may serve as a potential therapeutic approach for controlling coronavirus-induced diseases.

### DMV formation by Nsp3, Nsp4, and Nsp6

During virus replication, coronaviruses form a double-membrane compartment by hijacking the host endoplasmic reticulum (ER) membrane and construct a ‘shelter’ where the viral components can escape cytosolic PRR sensing^14^. Several coronavirus nonstructural proteins, such as Nsp3, Nsp4, and Nsp6, are known to play pivotal roles in double-membrane vesicle (DMV) formation. Nsp4, along with Nsp3 and Nsp6, hijacks the ER membrane and forms DMVs by inducing membrane rearrangement. Viral replicase complexes that contain PAMP RNA species are then sequestered into the DMVs, thus preventing activation of cytosolic RNA sensors^78^.

### Nsp5

Together with Nsp3, Nsp5 is responsible for processing viral polyproteins through its 3C-like protease activity and contributes to virus replication. Intriguingly, porcine deltacoronavirus (PDCoV) Nsp5 has been shown to suppress host antiviral responses by processing host antiviral proteins, such as STAT2^79^, NF-kappa-B essential modulator (NEMO)^80^, and mRNA decapping protein 1a (DCP1a)^81^. Given that the inhibitory effect of Nsp5 is dependent on protease activity, protease inhibitors specifically targeting Nsp5 may be a new therapeutic approach.

### Nsp8

Nsp8, together with Nsp7, are components of the viral RdRp complex and play a role in facilitating RdRp activity^82^. In addition, a recent result from the preprint manuscript suggested that SARS-CoV-2 Nsp8 also plays an inhibitory role in host innate immunity. It was shown that Nsp8 could directly bind to MDA5 CARD and block K63-linked polyubiquitination, thereby suppressing the MDA5-derived IFN signaling pathway^83^.

### RNA modification by Nsp13, 14, 15, and 16

Coronavirus genomes encode multiple RNA-modifying enzymes that alter the biochemical properties of both host and viral RNAs. Via these enzymes, coronaviruses have evolved several means to evade the host immune system by modifying their RNAs to avoid being sensed by antiviral receptors. It has been shown that coronavirus Nsp13, Nsp14, and Nsp16 are associated with RNA modification. Nsp13 of various coronaviruses was shown to regulate the removal of the 5′-ppp moiety from viral RNAs, a molecular signature of RIG-I ligand^10,84,85^.

The addition of a cap structure to the 5′-end of viral RNA by coronavirus capping enzymes is a smart strategy. For example, SARS-CoV Nsp14 plays a pivotal role in the 5′-capping of viral mRNA via its guanine-N7-methyltransferase activity (N7-MTase)^12,86^. Furthermore, Nsp16 of SARS-CoV^11^ and SARS-CoV-2^87^ have been shown to regulate the 2′-O-methylation of viral RNA, which is essential for 5′ capping, thereby avoiding activation of antiviral sensors such as MDA5 and IFIT family proteins^27^.

In addition to regulating 5′ capping, coronaviruses possess another strategy to modify viral RNA by viral endoribonuclease. As described in the previous section, recent studies have elucidated that Nsp15 can eliminate the 5′-polyuridine region from the virus PUN RNA to prevent being sensed by cytosolic dsRNA sensors, including MDA5, PKR, and OAS/RNase L^42^. Therefore, these coronavirus enzymes involved in RNA modification play a critical role in blocking the early events of host antiviral sensing.

### ORF3a

ORF3a localizes to the plasma membrane of the ER and Golgi and induces ER stress by activating the PKR-like ER kinase (PERK) pathway. The activated PERK pathway can induce the phosphorylation and cause the degradation of IFNAR1^88^, evading host antiviral IFN programs (Fig. 3). In addition, ORF3a also regulates apoptotic pathways. Recent studies have shown that ORF3a of SARS-CoV and SARS-CoV-2 can trigger host cell apoptosis by inducing caspase activation or Golgi fragmentation^89,90^, indicating that ORF3a targets multiple cellular pathways to hamper host antiviral responses.

On the other hand, ORF3a appears to be involved in activating inflammatory pathways. It has been reported that SARS-CoV ORF3a can induce NLRP3-dependent inflammasome activation by directly interacting with ASC^91^. Moreover, ORF3a upregulates the NF-κB pathway by interacting with TRAF3, contributing to the inflammasome pathway. However, it is unclear whether ORF3a-mediated proinflammatory responses are beneficial for the viral life cycle.

### ORF3b

ORF3b was shown to have a unique shuttling behavior, translocating from the nucleus to the outer membrane of mitochondria. Localization of SARS-CoV ORF3b in mitochondria is critical for its inhibition of the MAVS-mediated IRF3 activation pathway, leading to reductions in IFN and ISG expression^92^. In addition, a recent report showed that SARS-CoV-2 ORF3b inhibits the IFN signaling pathway by blocking the nuclear translocation of IRF3. Interestingly, SARS-CoV-2 produces a smaller ORF3b than SARS-CoV with a shortened C-terminal region. Further analysis performed with several ORF3bs from various CoV strains showed that ORF3b with a shorter C-terminus region showed a more potent inhibitory function than its longer form. Notably, the cytoplasmic localization of ORF3b seems to be associated with its antagonism of the IFN signaling pathway. The authors found that the longer ORF3b found in SARS-CoV-related strains possesses a putative nuclear localization signal (NLS) in its C-terminal region and facilitates its nuclear localization. However, shorter ORF3b from SARS-CoV-2-related strains is predominantly localized in the cytoplasm due to the loss of NLS in the shortened C′ terminus region, resulting in stronger antagonistic function^93^. However, the underlying mechanism of SARS-CoV-2 ORF3b-mediated inhibition of IRF3 nuclear translocation to the cytoplasm still needs to be clarified.

### ORF6

ORF6 is a small protein that is mainly localized in the cytoplasm and partially colocalized in the ER and Golgi compartment. Together with Nsp1, ORF6 appears to be the most potent antagonist of host antiviral responses. Multiple groups have shown that ORF6 can suppress IFN and ISG induction by blocking specific nuclear import and export pathways. For example, ORF6 inhibits the nuclear import of innate immune signaling-related transcription factors such as IRF3 and STAT1^70,71^, resulting in the shutdown of downstream events (Figs. 2 and 3). Mechanistically, ORF6 targets the karyopherin-containing importin complex through its acidic region of its C′ terminus to block the nuclear transport of NLS-containing proteins^94^. In addition, ORF6 was shown to block the nuclear export of newly synthesized mRNA by interacting with the nuclear pore complex (NPC) proteins Rae1 and Nup98, thereby suppressing gene expression in infected cells^95,96^.

### ORF7a

Studies have shown that SARS-CoV ORF7a induces cell cycle arrest and apoptosis via a caspase-dependent pathway^97^. ORF7a interacts with pro-survival factors, such as B cell lymphoma (Bcl)-2 and Bcl-X_L_, and directly inhibits the pro-survival function of Bcl-X_L_, triggering apoptosis^98^. Furthermore, ORF7a can directly bind to and inhibit bone marrow stromal antigen 2 (BTS-2)/Tetherin, an antiviral protein that restricts the release of enveloped viruses from host cells^99^.

In addition, ORF7a was shown to suppress protein translation by hampering the host stress response pathway. For example, ORF7a specifically targets the p38 MAPK pathway and inhibits cellular protein synthesis, resulting in apoptosis^100^. This inhibitory regulation can potentially cause a reduction in antiviral protein synthesis, leading to the pathogenesis of SARS-CoV.

### ORF9b

Although the initial study with SARS-CoV suggested that ORF9b is a virion-associated accessory factor, recent studies have further elucidated that ORF9b can inhibit host innate immunity by targeting mitochondria-mediated antiviral immunity. Shi et al.^101^ showed that SARS-CoV ORF9b could trigger the degradation of MAVS, TRAF3, and TRAF6 by tethering poly(rC)-binding protein 2 (PCBP2) and E3 ligase atrophin 1 interacting protein 4 (AIP4) in mitochondria, thereby suppressing downstream antiviral responses. A recent study with SARS-CoV-2 suggested that ORF9b antagonizes the type I IFN pathway through association with translocator of outer membrane 70 (TOM70), a critical mitochondrial import receptor regulating IFN responses^102^. Overexpression of TOM70 rescued IFN-β expression, further confirming the specific inhibition of the TOM70-mediated antiviral signaling pathway by ORF9b.

### Nucleocapsid

The nucleocapsid (N) protein is a coronavirus structural protein that plays essential roles in viral transcription and virion assembly. In addition to its contribution to virus replication, N plays pivotal roles in suppressing host innate immunity^103**–**105^. Two studies with SARS-CoV showed that N suppresses IFN production. Lu and colleagues suggested that SARS-CoV N targets the upstream event of RNA sensing to block the IFN signaling pathway^103^. A subsequent study by Hu and colleagues also showed that N could directly bind to tripartite motif-containing 25 (TRIM25) via its C-terminal region and interfere with the TRIM25–RIG-I interaction, thereby suppressing TRIM25-mediated RIG-I ubiquitination and activation^105^.

In addition to targeting the RNA sensing pathway, a recent study with SARS-CoV-2 provided evidence that SARS-CoV-2 N also targets the IFN signaling pathway by suppressing the phosphorylation and nuclear translocation of STAT1 and STAT2^104^ (Fig. 3). However, the exact mechanism underlying N-mediated inhibition of the IFN signaling pathway is still unclear. Nevertheless, these data suggest that the coronavirus N protein can hamper host innate immunity by targeting multiple antiviral responses.

### Membrane

The SARS-CoV membrane (M) protein was shown to blunt the formation of the TRAF3-TRAF family member-associated NF-kB activator (TANK)-TBK1/IKKε complex, thereby impeding downstream IRF3/IRF7 activation and IFN production^106^. Mechanistically, the TM1 (1–38 amino acids) region of the M protein is critical for its localization in the Golgi apparatus, where M interacts with innate immune proteins such as RIG-I, TBK1, IKKε, and TRAF3, blocking downstream antiviral signaling cascades^107^. Moreover, a recent study also showed that SARS-CoV-2 M suppresses type I and III IFN production. SARS-CoV-2 M could directly bind to essential molecules of the cytosolic viral RNA sensing pathway, such as RIG-I, MDA5, MAVS, and TBK1, and prevent their interaction^108^.

## Host-mediated immunopathogenesis

The immune system recognizes invasive pathogens, responds proportionally to the pathogen burden, and then must properly return to homeostasis. Although IFNs and cytokines are essential for antiviral immunity, aberrant immune responses by the dysregulated host immune system can cause severe inflammatory conditions. The detrimental physiological status that occurs via excessive host immune responses is termed a ‘cytokine storm’^109^. Various pathological conditions, such as pathogen infections, cancers, autoimmune conditions, and immunotherapies, are involved in triggering the primary host immune response. Subsequently, abnormally controlled immune responses against those primary causative agents trigger secondary systemic inflammation that leads to the onset of a cytokine storm. The typical pathophysiological outcome induced by a cytokine storm includes hyperactivation of immune cells, abnormal blood cell counts, and increased levels of circulating cytokines, such as tumor necrosis factor (TNF)-α, type I and II IFNs, interleukin (IL)-1, IL-6, IL-12, IFN-γ-induced protein-10 (IP-10), or monocyte chemotactic protein-1 (MCP-1), ultimately causing multiorgan failure and death^109^.

Both innate and adaptive immune cells act as pathological cellular or cytokine drivers. Innate immune cells, such as neutrophils, macrophages, and natural killer (NK) cells, are most often implicated in the pathogenesis of a cytokine storm. Hyperactivated neutrophils can cause severe tissue damage by uncontrolled secretion of reactive oxygen species (ROS) and cytokines through releasing granules and neutrophil extracellular traps (NETs)^110^. In many cases of cytokine storms, macrophages produce excessive amounts of cytokines, including IFN-γ, TNF-α, IL-1, and IL-6, contributing to tissue damage and diminishing the cytolytic function of NK cells, which can lead to prolonged antigenic stimulation and perpetuating inflammation^111**–**113^. In addition, activation of effector T lymphocytes is closely associated with the pathogenesis of cytokine storms. In particular, type I helper T cells (Th1) are the predominant cells driving robust proinflammatory conditions. The production of large quantities of IFN-γ by Th1 cells can induce exaggerated inflammatory reactions by activating multiple innate immune cells. Notably, recent studies have shown that elevated levels of IL-17 produced by Th17 cells are also involved in the pathogenesis of the cytokine storm in COVID-19 patients^114,115^.

Highly pathogenic coronaviruses, such as SARS-CoV, MERS-CoV, and SARS-CoV-2, often cause aberrant host immune responses, resulting in imbalanced production of IFNs and proinflammatory cytokines. Patients with serious illness caused by these viruses often show symptoms of a cytokine storm characterized by highly concentrated proinflammatory cytokines and chemokines in the plasma, leading to septic shock, acute respiratory distress syndrome (ARDS), and multiple organ failure^116**–**118^. Notably, patients with SARS-CoV- and SARS-CoV-2-associated cytokine storms showed a unique immunopathogenesis. For example, although lymphopenia is not frequently observed in cytokine storm disorders, reduced numbers of T lymphocytes and NK cells are a hallmark of severe SARS and COVID-19^119^. However, there is no clear explanation for the underlying mechanism involved in the unique immunopathogenesis induced by certain coronaviruses.

Although the role of immune dysregulation and the cytokine storm in COVID-19 remains unknown, recent studies have provided one possible link for the onset of SARS-CoV-2-associated hyperinflammation. Neuropilin-1 (Nrp1) is another cell entry coreceptor of SARS-CoV-2^120,121^. Given that Nrp1 is expressed in regulatory T (Treg) cells and stabilizes Tregs^122^, professional immune suppressor cells, it is reasonable to hypothesize that SARS-CoV-2 may infect Treg cells through Nrp1, thereby reducing the Treg population or function and leading to uncontrolled host proinflammatory responses. Intriguingly, two case studies from COVID-19 patients have shown that the treatment of critically ill ARDS patients with Treg-based therapy resulted in recovery from the disease^123^. Thus, targeting the Nrp1-mediated SARS-CoV-2 entry pathway may serve as a potential therapeutic approach to control COVID-19.

## Conclusion and future perspectives

Since the beginning of the 2000s, outbreaks of betacoronaviruses have continually occurred approximately every ten years. Today, we are facing an unprecedented public health threat and world economic crises due to the global outbreak of SARS-CoV-2, another betacoronavirus. Although it has been almost one year since SARS-CoV-2 was first reported, the virus is still spreading rapidly on the globe, exponentially creating large patient populations.

What makes these viruses highly pathogenic and deadly? There are distinct features of these coronaviruses that are associated with viral virulence and pathogenicity. First, all three highly pathogenic betacoronaviruses are zoonotic and thought to originate from bats or pangolins^124^. Thus, our immune system is ‘naïve’ and has not been prepared for ‘never-before-seen’ invaders. Second, as we discussed intensively, betacoronaviruses possess multiple mechanisms targeting various innate immune responses to evade host antiviral defense programs. Failure of timely and appropriate innate immune activation may lead to robust viral propagation and is directly associated with disease severity and mortality. Last, coronaviruses can cause elevated levels of circulating proinflammatory cytokines and chemokines via uncontrolled host immune responses, termed the cytokine storm.

The main measures currently in effect are preventing the spread of COVID-19 by limiting the movement of people and maintaining a social distance from others. However, these are only symptomatic measures and do not provide a fundamental solution. Current healthcare systems treat patients with limited respiratory management using existing treatments used for other diseases. However, the mechanism of action of the existing therapies being used for the treatment of the diseases is not clear and needs to be elucidated. Thus, understanding the underlying immune escape mechanisms of coronaviruses is essential for developing specific treatments for coronavirus-derived diseases, such as COVID-19.

Since coronaviruses possess multiple immunosuppressive mechanisms and can cause excessive immune responses by abnormal activation of the complex host immune system, targeting a single viral factor may not be effective for controlling viral pathogenesis. Therefore, either preventing the onset of a viral infection by reducing the chance of being in contact with a potential reservoir of zoonotic origin or protecting against virus-induced diseases through effective vaccination would be ideal for avoiding an unprecedented disaster. A continuous effort to understand the underlying mechanisms of host-virus interactions is necessary to overcome the current pandemic of COVID-19 and prepare for the potential pandemic that may occur in the future.
